# Treatment Advances in Tumor-Induced Osteomalacia

**DOI:** 10.1007/s00223-024-01317-x

**Published:** 2025-01-04

**Authors:** Iris R. Hartley, Kelly L. Roszko

**Affiliations:** National Institute of Dental and Craniofacial Research, NIH, Bethesda, MD 20892 USA

**Keywords:** Tumor-induced osteomalacia, Hypophosphatemia, Treatment, Mineral metabolism

## Abstract

Tumor-induced osteomalacia (TIO) is a rare paraneoplastic syndrome caused by hypersecretion of fibroblast growth factor 23 (FGF23) by typically benign phosphaturic mesenchymal tumors (PMTs). FGF23 excess causes chronic hypophosphatemia through renal phosphate losses and decreased production of 1,25-dihydroxy-vitamin-D. TIO presents with symptoms of chronic hypophosphatemia including fatigue, bone pain, weakness, and fractures. Definitive treatment is surgical resection of the PMT with wide margins. Other therapeutic options are necessary when the tumor is unable to be localized, not amenable to complete resection, or when the patient is not a good surgical candidate. Alternative ablative approaches such as radiotherapy, radiofrequency ablation, and cryoablation, have been used with variable success and limited follow up. Medical management is warranted both prior to definitive therapy and in non-operable cases to improve symptoms and allow for bone remineralization. Oral phosphate and calcitriol were the mainstay of medical therapy, however, the development of burosumab, a monoclonal blocking antibody to FGF23, has introduced an approved therapy that improves hypophosphatemia and symptoms in patients with TIO. In select cases, cinacalcet can be an effective adjuvant to phosphate and calcitriol. Continued monitoring for tumor growth is necessary while on medical therapy. Infigratinib, a selective FGFR tyrosine-kinase inhibitor targeting a causative tumoral fusion protein, can reverse the biochemical findings of TIO and possibly reduce tumor mass; however, its use is constrained by serious side effects. Overall, innovations in medical and interventional treatments have broadened therapeutic options for patients with PMTs, particularly in cases where a curative surgical resection is not possible.

## Introduction

Tumor-induced osteomalacia (TIO) is a rare paraneoplastic syndrome in which dysregulated secretion of fibroblast growth factor 23 (FGF23) from a tumor causes hypophosphatemia primarily due to renal phosphate wasting. TIO is typically caused by a phosphaturic mesenchymal tumor (PMT), which is a distinct histologic diagnosis [1]. PMTs are usually benign and can be found anywhere in the body in soft tissue or bone [2]. Rarely, non-PMT solid or hematologic malignancies may also produce excess FGF23. While often labelled TIO, this syndrome may represent a distinct pathological entity, sometimes referred to as cancer-associated osteomalacia (CAO) [3].

FGF23 is a 251 amino acid protein important in maintaining phosphate homeostasis. At the renal proximal tubules, FGF23 binds to fibroblast growth factor receptor 1 (FGFR1) and the co-receptor α-klotho to downregulate the sodium phosphate cotransporters NaPi-2a and NaPi-2c, which induces phosphaturia by decreasing the renal reabsorption of phosphate [4, 5]. Additionally, FGF23 signaling decreases the 1-α-hydroxylation of 25-hydroxy vitamin D, decreasing levels of active 1,25(OH)_2_-vitamin D [5], and thereby reducing intestinal absorption of calcium and phosphate.

The presenting clinical features of TIO are those of chronic hypophosphatemia including muscle weakness, fatigue, bone pain, fractures, and deformity. Characteristic biochemical findings include hypophosphatemia, renal phosphate wasting, as indicated by a low calculated tubular reabsorption of phosphate (TRP) or low tubular maximum phosphate reabsorption rate (TmP/GFR), low or inappropriately normal 1,25(OH)_2_-vitamin D, and elevated or inappropriately normal FGF23 levels [6–8]. Secondary or tertiary hyperparathyroidism often develops due to both low 1,25(OH)_2_-vitamin D levels and the PTH-stimulating effect of phosphate supplementation [6, 9]. Elevated alkaline phosphatase is frequently present reflecting chronic osteomalacia and/or fractures.

**Fig. 1:**
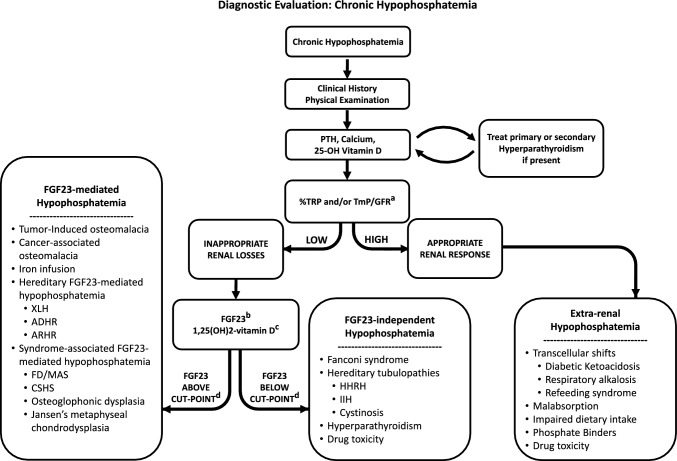
Diagnostic evaluation for chronic hypophosphatemia. ^a^%TRP and TmP/GFR should ideally be measured when the patient is not taking phosphate supplementation. ^b^Intact FGF23 assays are preferable over c-terminal FGF23 assays when available [7]. ^c^Low or inappropriately normal levels of 1,25(OH)_2_-vitamin D are suggestive of FGF23 excess, and may serve as a surrogate indicator when FGF23 measurement is unavailable. However, caution is needed when interpreting these levels, as 1,25(OH)_2_-Vitamin D may also be influenced by PTH and direct proximal renal tubular damage. ^d^The normal range for FGF23 often reflects FGF23 levels when phosphate is in the normal range. Specific cut-points have been defined for distinguishing FGF23-mediated from FGF23-independent causes of hypophosphatemia [7]. *%TRP* %tubular reabsorption of phosphate, *TmP/GFR* tubular maximum reabsorption of phosphate, *XLH* X-linked Hypophosphatemia, *ADHR* Autosomal dominant hypophosphatemic rickets, *ARHR* autosomal recessive hyphophosphatemic rickets, *FD/MAS* fibrous dysplasia/McCune-Albright syndrome, *CSHS* cutaneous skeletal hypophosphatemia syndrome, *HHRH* Hereditary hypophosphatemic rickets with hypercalciuria, *IIH* idiopathic infantile hypercalcemia

Determining the cause of chronic hypophosphatemia presents a considerable clinical challenge, and thorough evaluation is necessary for accurate diagnosis (Fig. 1). FGF23-mediated hypophosphatemia in TIO may be confused with hyperparathyroidism, renal tubulopathies, malabsorption, drug toxicity, or transcellular shifts. Moreover, other causes of FGF23-mediated hypophosphatemia share identical biochemical features of TIO and should be ruled out prior to making the diagnosis. These include genetic conditions such as X-linked hypophosphatemia (XLH), autosomal dominant hypophosphatemic rickets (ADHR) and autosomal recessive hypophosphatemic rickets (ARHR), as well as iatrogenic factors such as iron infusion, particularly of ferric carboxymaltose [10, 11]. Other syndromes can also be associated with FGF23-mediated hypophosphatemia, including fibrous dysplasia/McCune-Albright syndrome (FD/MAS), cutaneous skeletal hypophosphatemia syndrome (CSHS), neurofibromatosis type 1, osteoglophonic dysplasia, and Jansen’s metaphyseal chrondrodysplasia [12–16].

Once the diagnosis is made, a major therapeutic challenge in TIO is localizing the tumor. This can be notoriously difficult as the tumors can be found anywhere in the soft tissue or bone [2, 17–22]. TIO tumors are often small, with a median tumor size of 2.7 cm [20]. Medical history and careful physical exam may help identify a possible tumor, although in the majority of cases, functional imaging is required for localization. Somatostatin receptors have been identified on the surface of the vast majority of PMTs [23] permitting their imaging with radio-labeled somatostatin analogs, specifically by DOTATATE/DOTATOC-PET/CT [24–28]. Fluorodeoxyglucose positron emission topography may also be informative [24]. Functional imaging should include the whole body from the head to the toes.

Once a lesion is identified on functional imaging, anatomic imaging is important to characterize the presumptive lesion and evaluate the possibility of surgical resection. In cases of multiple positive findings, or in cases where surgical resection would be complicated, selective venous catheterization can confirm the presence of the PMT prior to intervention [2, 29]. Biopsy of the lesion should be avoided since seeding from the tumor may lead to increased tumor recurrence [30].

## Surgical and Procedural Treatments

The only definitive curative treatment for TIO is complete surgical resection of the tumor with wide margins [3, 17, 31, 32]. Large systemic reviews have reported that more than 85% of TIO tumors can be localized, and 90% of these can be successfully removed with surgery [20, 33]. PMTs are not encapsulated and may infiltrate into soft tissue and along boney trabeculae so wide excision is imperative [34]. One study of 230 patients with TIO showed that following surgery, 11.3% of tumors persisted and 7.0% recured with a median time to recurrence of 33 months. Tumors of the spine had the highest failure rate in achieving surgical cure (77.8%) [35]. When the margins of the surgical specimen are positive, there is a higher likelihood for recurrence [36]. Curettage of bone lesions also results in a higher rate of recurrent or persistent disease than wide excision [37].

Upon surgical removal of the PMT, the biochemical abnormalities of TIO rapidly correct. Intact FGF23 (iFGF23) levels quickly decrease while the C-terminal FGF23 (cFGF23) levels decrease at a slower rate, leading to an elevated cFGF23/iFGF23 ratio [2]. 1,25(OH)_2_-Vitamin D levels increase and often exceed the upper limit of normal. Blood and urine phosphate typically return to the normal range within 5 days [2, 38]. It may take as long as a year for osteomalacia to heal and alkaline phosphatase levels to normalize, during which supplemental calcium may be necessary to avoid secondary increases in PTH [3]. Colangelo et al. showed significant improvement in bone mineral density and trabecular microarchitecture by DXA in the lumbar spine, femoral neck, and total hip which peaked at 26.7 ± 6.5 months post-surgery [39]; however, post-operative short-term losses in the distal radius and tibia have also been reported which might be due to the effect of increased PTH on these sites [40]. If biochemical parameters do not improve following surgery, the workup should be re-initiated to search for persistent or metastatic disease [31]. Even after successful resection, phosphate should be measured yearly to monitor for recurrence [16].

In cases where surgical resection is not possible, external beam radiation [41], radiofrequency ablation [42, 43], and cryoablation [44–46] have been used with some success, but long-term efficacy of these procedures have yet to be established. Although peptide receptor radionuclide therapy (PRRT) with 177-Lutetium DOTATATE or DOTATOC could theoretically target PMTs directly by taking advantage of the somatostatin receptors expressed in most TIO tumors, it failed to achieve a lasting remission in six patients with TIO [11, 47–50]. Considering the lack of demonstrated long-term efficacy of alternative interventions, when possible, surgical removal with wide margins is still considered the best option for cure.

## Medical Management

Following confirmation of the diagnosis of TIO, medical therapy should be initiated as soon as possible to reduce symptoms and strengthen bones in preparation for surgery [16]. Chronic medical treatment is necessary in cases where a PMT is either non-localizable or inoperable, or a patient is a poor surgical candidate [51].

Until recently, the primary medical approach to managing TIO involved using phosphate salts and activated vitamin D (calcitriol, alfacalcidol) to increase phosphate levels and relieve clinical symptoms. The required dose of these medications can vary widely and should be adjusted based on the individual’s symptoms and laboratory values [3, 31]. Frequent, smaller doses of phosphate are preferred to minimize gastrointestinal side effects, although this can be burdensome and challenging for patients. Phosphate monotherapy should be avoided, as it frequently leads to secondary hyperparathyroidism, exacerbating hyperphosphaturia and worsening hypophosphatemia [16]. Furthermore, chronic secondary hyperparathyroidism may progress to tertiary hyperparathyroidism [52]. To prevent secondary hyperparathyroidism, it is essential to combine phosphate with calcitriol or alfacalcidol, although excessive activated vitamin D supplementation can cause hypercalciuria, increasing the risk for nephrolithiasis, nephrocalcinosis, and renal impairment [52]. Oral calcium supplementation may also be required to allow for bone remineralization but must be taken separately from phosphate supplementation, further complicating therapy. This regimen requires close monitoring, targeting low or low-normal blood phosphate levels, and both intact parathyroid hormone (PTH) and 24-h urine calcium excretion levels within the normal range. The goal of treatment is improvement in bone quality which is reflected by normalization of alkaline phosphatase [3].

The calcimimetic cinacalcet, an agonist of the calcium-sensing receptor, may be used as an adjuvant treatment along with calcitriol and phosphate in select cases. This approach takes advantage of the finding that FGF23’s action on renal phosphate excretion is PTH-dependent [53]. By decreasing PTH, cinacalcet decreases the effect of FGF23 on the renal tubule, resulting in decreased renal phosphate losses. Two patients with TIO were successfully treated with cinacalcet, as evidenced by increased blood phosphate and decreased calcitriol and phosphate supplement requirements [54]. Based on similar reasoning, a patient with TIO and tertiary hyperparathyroidism underwent a deliberate total parathyroidectomy, resulting in improved blood phosphate levels and decreased phosphate requirements despite persistently elevated FGF23 [55]. With both approaches, the reduction in PTH has the potential of increasing the risk of hypercalciuria and hypocalcemia, necessitating careful monitoring [54]. More studies are needed to establish the optimal use of these options in TIO.

Burosumab, a human monoclonal antibody against FGF23, has emerged as a promising new treatment option for patients with unresectable or non-localizable disease. It has been approved for the treatment of TIO in the USA, EU, China, and Japan [16, 56–58]. In two open-label phase 2 studies, a total of 27 adults with TIO were effectively treated with burosumab, showing improvements in phosphate levels, fracture healing, and quality of life [57]. Average serum phosphate increased and was maintained in the normal range up to 144 weeks [56, 57]. At week 144, 33% of 249 fractures and pseudofractures in 14 patients were fully healed and 13% were partially healed [57]. Within the confines of the trials, blood phosphate was maintained in the low-normal range, so fracture healing may be more substantial at higher dosages in clinical practice [59]. After 96 weeks, bone mineral density was improved at the lumbar spine and total hip in 12 subjects [56]. While on burosumab, the bone turnover markers type 1 collagen C-telopeptides (CTX), procollagen 1 N-terminal propeptide (P1NP), bone specific alkaline phosphatase (BALP), and osteocalcin (OC) increased initially, peaked at week 16 or 24 and then started to trend down [56, 57]. In a subset of 8 patients who participated in exit interviews, patients reported symptomatic improvements including in physical functioning and mental well-being [60]. Additional trials and case reports have supported the ability of burosumab to improve serum phosphate levels, pain, and physical performance in patients with TIO [56, 61, 62]. Data regarding the long-term safety and impacts of burosumab are needed.

Burosumab is given as a subcutaneous injection which is administered every 2 to 4 weeks up to a maximum of 2 mg/kg/dose. Adjustments are made based on the peak fasting serum phosphate level obtained midway between doses. Blood phosphate, creatinine, alkaline phosphatase, intact PTH, and blood and urinary calcium should be monitored while on therapy [16]. Due to limitations of existing FGF23 assays, which cannot differentiate between burosumab-bound and free FGF23, FGF23 levels are uninterpretable while on burosumab [63]. Given the long half-life of burosumab of 16.4 days [64], it may thus be advisable to delay burosumab initiation until there is a confirmed diagnosis of TIO and it is certain that venous sampling will not be needed. This limitation also prevents FGF23 from being used as a reliable marker of disease progression or tumor growth while a patient is taking burosumab.

It is important to note that treatment with either burosumab or the combination of phosphate and activated vitamin D does not remove the underlying causative tumor, and local expansion or metastatic extension is still possible [3]. Thus, attempts to localize the tumor or monitor growth should continue with imaging every 1–2 years even while patients are on medical therapy [16].

Despite the presence of somatostatin receptors on most PMTs, octreotide therapy did not result in meaningful changes in blood phosphate, FGF23, or TRP levels in five patients with TIO [65].

To molecularly target tumors directly, infigratinib, an orally bioavailable FGFR1-3 tyrosine kinase inhibitor, has been investigated for treatment of TIO. The rational for using infigratinib arises from the frequent occurrence of a presumptively causative gene fusion of fibronectin 1 and FGFR1 (FN1-FGFR1) which has been identified in over 40% of cases of resectable PMTs [66]. When used in a patient with metastatic TIO harboring a FN1-FGFR1 fusion, infigratinib was shown to profoundly lower FGF23 levels and reduce tumor burden, although the biochemical response reversed after drug discontinuation [67]. Intriguingly, evidence of apparent metaplastic differentiation into mature lamellar bone was observed in the patient’s tumors. These findings prompted a follow up study in four patients with benign TIO in which infigratinib effectively reversed the biochemical abnormalities of TIO, including normalization of phosphate, intact FGF23, and 1,25(OH)_2_-Vitamin D levels. Upon cessation of the drug after up to 24 weeks of therapy, however, biochemical parameters returned to baseline, and no patients experienced prolonged remission. Further enrollment was halted due to a high incidence of ocular AE’s, including corneal keratitis and scarring [68, 69]. While effective in correcting the biochemical abnormalities of TIO and possibly also impacting tumor growth, a short course of infigratinib failed to induce a lasting remission, and long-term therapy is limited by serious ocular side effects. Still, it may be an option to consider in the treatment of rare, life-limiting, metastatic disease. Advances in targeted treatments that more selectively block FGFR1 may permit the use of higher doses and more sustained treatment without side effects.

In cases of CAO, in which FGF23 excess is caused by non-PMT solid or hematologic malignancies, the treatment approach involves targeting the underlying malignancy, while using medical therapies to manage the hypophosphatemia and symptoms. The use of burosumab in treating CAO is likely beneficial in select cases, but its efficacy has not been confirmed.

## Conclusions

TIO, a rare disorder of excess FGF23 secretion, can cause debilitating muscle weakness, fatigue and fractures. While surgical resection of the causative tumor with wide margins remains the best definitive treatment, recent innovations in medical therapy, such as burosumab and infigratinib, and procedural treatments, including radiotherapy and cryoablation, have improved the options for patients with TIO.
